# Respiratory functions and health risk assessment in inhalational exposure to vinyl acetate in the process of carpet manufacturing using Monte Carlo simulations

**DOI:** 10.1007/s11356-022-24469-5

**Published:** 2022-12-05

**Authors:** Amir Hossein Khoshakhlagh, Hamid Reza Saberi, Agnieszka Gruszecka-Kosowska, Vikas Kumar

**Affiliations:** 1 Department of Occupational Health Engineering, School of Health, Kashan University of Medical Sciences, Kashan, Iran; 2 Department of Environmental Protection, Faculty of Geology, Geophysics, and Environmental Protection, AGH University of Science and Technology, Al. Mickiewicza 30, Krakow, 30-059 Poland; 3 Environmental Engineering Laboratory, Departament d’ Enginyeria Quimica, Universitat Rovira i Virgili, Av. Països Catalans 26, 43007 Tarragona, Catalonia Spain; 4 IISPV, Hospital Universitari Sant Joan de Reus, Universitat Rovira i Virgili, Reus, Spain

**Keywords:** Vinyl acetate, Persian carpets, Occupational exposure, Human health risk assessment, Pulmonary function, Monte Carlo simulation

## Abstract

Vinyl acetate (VA) is a volatile compound and the main compound of the carpenter’s glue. VA causes upper respiratory tract irritation, cough, and hoarseness in occupational exposure. As Iran is one of the biggest carpet producers in the world, this study was carried out to determine the inhalational health risk for employees exposed to VA. To the best of our knowledge, this was the first health risk assessment and the first evaluation of the lung functions and respiratory symptoms in employees exposed to VA. In the six finishing shops of carpet manufacturing industry in Kashan city, Iran the cross-sectional studies were conducted in 2022. The subjects comprised of forty male employees exposed to VA and of forty non-exposed employees in the reference group. VA analyses in the workers’ breathing zones were performed based on the National Institute for Occupational Safety and Health (NIOSH) 1453 Method. VA concentrations were measured using Gas Chromatography-Mass Spectrometry (GC–MS). Inhalational risk assessment to VA was performed using the United States Environmental Protection Agency method and the Monte Carlo simulations. Respiratory functions were determined using the spirometry indices. In the exposed employees, considerably higher prevalence rates of pulmonary symptoms were observed in comparison with the control group. Statistical analysis showed a remarkable difference between lung function parameters measured in the case and the control groups. The VA Hazard Quotient (HQ) values for all working posts, except the quality control unit, were > 1 indicating the substantial inhalational non-cancerogenic risk. The sensitivity analysis revealed that the VA concentrations and exposure time had the most significant contribution in the uncertainty assessment. Therefore, it is recommended to decrease exposure to VA concentrations and to reduce the working time of exposed employees.

## Introduction

Managing hazardous substances in industrial processes is one of the most critical stages in the life cycle of chemicals. Exposure to chemicals in the workplaces can pose irreparable adverse effects to employees’ health. Exposure to chemical compounds may cause a variety of health effects depending on physico-chemical properties of chemicals, route of exposure (inhalation, contact with skin and eyes, and ingestion), their toxicity described by mode of action (MoA) and absorption, distribution, metabolism, and excretion (ADME) processes in the receptor, individual differentiation among exposed populations, the extent and duration of exposure (WHO 2000; Engelen et al. 2007; Leeuwen 2007).

Nowadays, occupational exposure is separately regulated from outdoor (WHO 2000) and indoor (WHO 2010) air quality requirements. In the European Union (EU), occupational exposure legislations are provided by the European Chemicals Agency (ECHA). The most relevant in the context of described research are the Chemical Agents Directive (CAD) and the Carcinogens and Mutagens Directive (CMD), both introducing minimum requirements for the protection of workers from the effects of chemical substances by setting occupational exposure limits (OELs) and from exposure to carcinogens and mutagens, respectively. In the USA, the Department of Labor ensures the safety of the workers through the Occupational Safety and Health Administration (OSHA) by adhering to the allowable chemical contents in workplaces described as for example: Permissible Exposure Limits (PELs), Recommended Exposure Limits (RELs), or Threshold Limit Values (TLVs). However, as there is no global unification, these regulations might vary among individual countries or even may not exist at all. This is specifically important in the industrial activities in the low income countries (LaDou et al. 2018).

Iranian carpets and rugs, also known as Persian, due to their color, pattern, and weaving style are famous globally and are being imported to various countries since the first century BC (Ateş 2022) and still are in high demand. In 2018–2019 about 55,500 tons of machine-woven carpets were exported from Iran, ranking the country the fifth biggest exporter in the world (Kohan Textile Journal 2020).

Vinyl acetate (VA) (CAS Registry Number 108–05-4) is a synthetic organic ester with the chemical formula C_4_H_6_O_2_, and the molar mass of 86.09 g mol^−1^ (PubChem). VA is a colorless volatile liquid with sweet and fruity odor. This compound is used as the monomer in the preparation of adhesives, paints, paper coatings, and textile finishes. In finishing shops of the carpet manufacturing industry, VA is the main ingredient of polyvinyl acetate (PVA), known as carpenter’s glue. The workers of carpet manufacturing industry’s finishing shops are potentially exposed to VA vapors (ATSDR 1992; Luttrell 2013) through inhalation, skin contact, and eyes irritation. Vinyl acetate was listed as one of the 189 hazardous air pollutants by the US Clean Air Act due to toxic effects of this compound (WHO 2010). Eye and upper respiratory tract irritations have been reported in case of acute exposure to this compound. Upper respiratory tract irritation, cough, and/or hoarseness have also been reported in chronic exposure to VA (ATSDR 1992; Bogdanffy et al. 1999; USEPA 1999; Luttrell 2013). No information is available on the reproductive, developmental, or carcinogenic effects of VA in humans (USEPA 1999), however, an increased incidence of nasal cavity tumors has been observed in rats exposed by inhalation (ATSDR 1992). In accordance, the International Agency for Research on Cancer (IARC) (IARC 1995) and the Japanese Society for Occupational Health (JSOH) (JSOH 2004) concluded VA to be possibly carcinogenic to humans (Group 2B) (EFSA 2010).

The health risk assessment is a procedure of identifying and quantifying the potential of adverse health effects to occur after exposure of the human population to environmental hazards. In the last version of the standard safety and health management system, occupational risk assessment is defined as a tool for determining the risks posed by hazards in workplaces by considering existing control measures and ensuing decisions whether the assessed risk value is at the acceptable level or not (Engelen et al. 2007; Leeuwen 2007; HSE 2014). By performing the risk assessment, the organization is capable to rate risks based on their risk potential, as well as to invest organization’s time and resources to focus on the factors being the most important in risk ranking and to neglect factors of neglible risk (WHO 2000; Engelen et al. 2007; Leeuwen 2007). In the chemical risk assessment, the level of risk for users is determined and the necessary measures are taken to protect personnel against hazardous compounds. Thus, risk analysis is one of the most important tool to maintain and improve the level of employees’ safety in the industry. Quantitative risk assessment procedure originally developed by the US Environmental Protection Agency (USEPA) (USEPA 1989) for chemicals is a method in which in the investigated exposure pathwys and for analyzed non-carcinogenic compounds risk rates are defined by the hazard quotient (HQ) values. In general, the HQ is defined as the ratio of the investigated compound’s exposure to a compound’s level that does not cause any adverse effects in the receptor organism (USEPA 1996; Engelen et al. 2007; Robson and Toscano 2007). In accordance, the target non-carcinogenic risk value was set to be equal to 1. It means that for HQ values < 1 the risk is acceptable or even neglible and for HQ values ≥ 1 the risk level cannot be accepted and corrective actions are required to lower the risk to the acceptable level (USEPA 2009).

Today, work-related respiratory illnesses are largely preventable. One of the important criteria in the diagnosis of lung diseases is the spirometric changes resulting during work shifts (Miller et al. 2005; Mahmood et al. 2010). Pulmonary function tests assess the extent of the disorder and damage to the lungs and the degree of lung disability; therefore, spirometry is of great value. Spirometry can indicate the onset of respiratory diseases in the early stages. Clinical examinations and periodic spirometry can prevent the increase of respiratory and pulmonary diseases in industrial workers. The most important and best parameters of pulmonary function include forced vital capacity (FVC), peak expiratory flow (PEF), forced expiratory volume in 1 s (FEV1) and FEV1/FVC ratio (Miller et al. 2005; Allen et al. 2008).

To the best of our knowledge, there were no investigations on estimating the human health risk to VA exposure in any industry before. Also, this is the first attempt to evaluate the lung function and respiratory symptoms in employees exposed to VA. Therefore, a comprehensive risk assessment of occupational exposure is highly required as it can contribute in preventing and reducing exposure to this chemical in the carpet finishing manufacturing industry.

The goal of the study was to determine the health risk for employees in carpet manufacturing industry in Kashan city, Iran related to exposure to VA. The objectives of the research were (1) human health risk assessment of VA in inhalational pathway during occupational exposure scenario; and (2) analysis of respiratory symptoms among employees exposed to VA using forced vital capacity (FVC), peak expiratory flow (PEF), forced expiratory volume in 1 s (FEV1), and FEV1/FVC ratio spirometry indices.

## Materials and methods

### Finishing process in the carpet manufacturing industry

The production process investigated in our research is described below. The pile of machine-made carpet in the surface-to-surface system is U-shaped, located around the carpet weave yarn. Since there are no knots in this category of carpets, the carpet can fall off pile due to the pulling force of the pile, so a kind of glue is used to strengthen the bases of the carpet. In addition to its role as a carpet pile, this glue is also considered as an effective layer in carpet braking and a factor resistant to carpet wear. In the machine-made carpet industry, this glue is called sizing behind the carpet, which is made of polyvinyl acetate (PVA). The carpets enter the brushing and beating (BB) unit and are brushed to remove excess yarn and lint. Then, the carpets reach the sizing (S) section. This part consists of three completely separate parts: (1) sizing pan: this part includes a large pan in which a cylinder rotates in the opposite direction of the carpet movement; the movement of the cylinder causes the sizing to be transferred from the pan to the under carpet; (2) dryer: after the carpets are impregnated with sizing material, the drying steps should be done. To do this in the finishing shops, the carpets are completed in two stages: in the warm room, glued carpets first enter the warm room. This part is usually heated by water vapor. Steam is transferred to special radiators through metal pipes. Under the radiators, strong fans are installed, which cause hot air to blow under the carpet and cause the sizing film to dry. The temperature in the first part of the room is about 60 °C, in the middle part about 80 °C and in the end part about 50 °C. The reason for adjusting the temperature in the mentioned order is that in the first stage, the carpet is prepared for heating, and in the second stage, the maximum temperature, where most of the drying of the film occurs, and in the third stage, by lowering the temperature, the carpet is ready to go out and accept the temperature outside the room; (3) steam cylinder (tambour): the carpet is wrapped on a very large roller with a diameter of 2.5 m, which is called tambour, after leaving the warm room. The working width is about 4 m. This large cylinder is heated by water vapor. The task of the tambour, which acts like a large steam iron, is to dry the sizing film by pressing it into the underlying tissue. This causes the possible wrinkles of the carpet to be removed and on the other hand, with the pressure applied to the carpet under the glue layer formed, it sinks into the porosity of the carpet and has a normal state under the carpet. Afterwards, carpet passes through the shearing (SH) machine and polishing its surface. Next, the machine-made carpet goes to embroidery, zigzag, and rooting (EZR) performed by sewing machines to increase the durability of the sides. Finally, the carpet quality control (QC) and rolling and packaging (RP) are done.

**Fig. 1:**
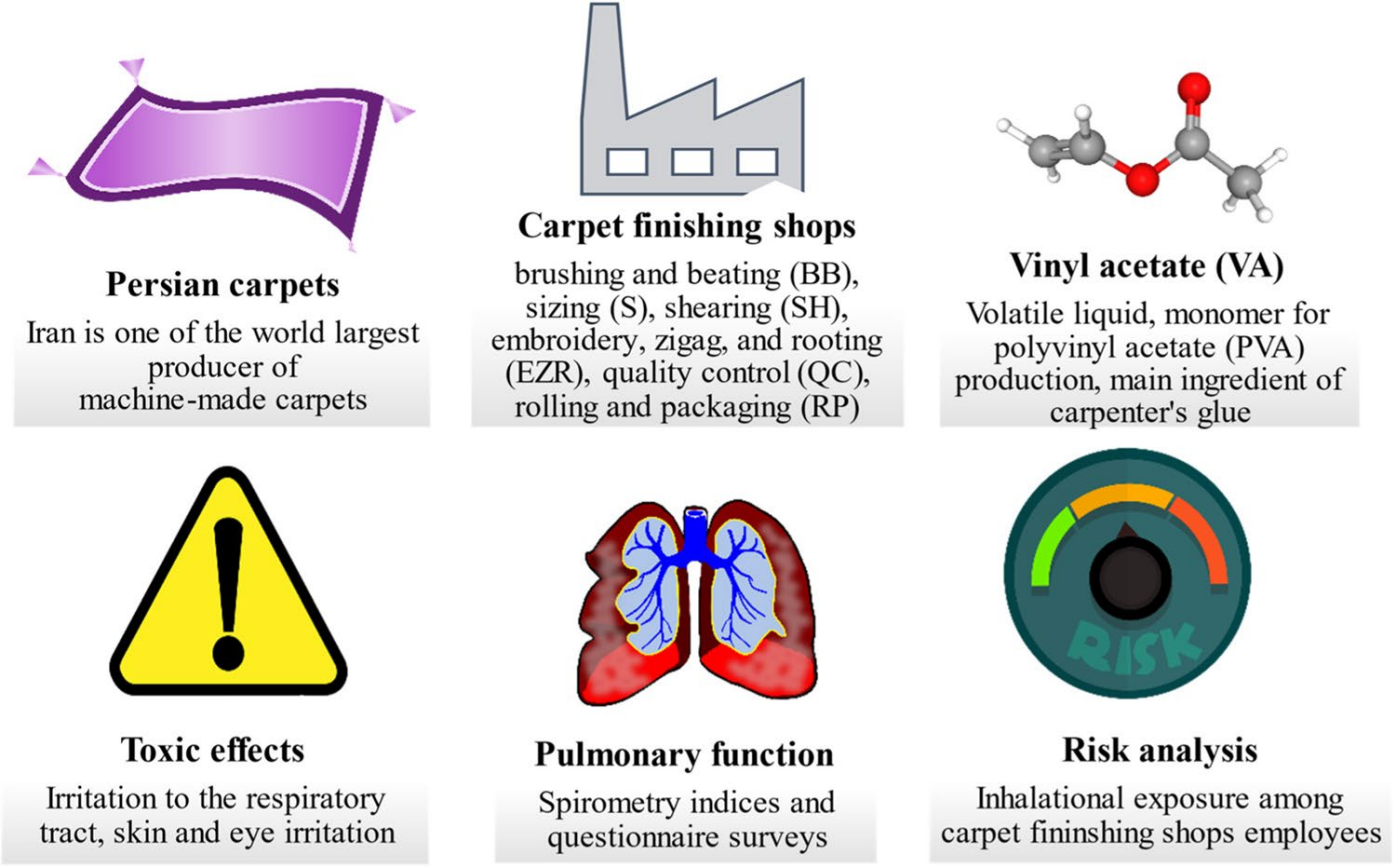
Scheme of the occupational exposure in finishing shops of carpet manufacturing industry (image sources: www.pubchem.ncbi.nlm.nih.gov; www.freeimages.com; www.pixabay.com)

Vapors of VA are generated in the finishing process when the raw materials including glue containing VA is poured and emptied into the glue injection boiler, additionally the vapors from the glue container of the sizing section and the glued carpets, as well as from the dryer, which re-evaporates VA owing to heat, emit it in the workplace, therefore, the workers are exposed to the emitted VA vapors. In the production sites investigated there were no industrial fumes extractors as well as the employees were not equipped in the personal protection equipment (PPE), i.e., masks. The illustrative scheme of the occupational exposure in the investigated finishing shops of carpet manufacturing industry is given in Fig. 1.

### Study population and questionnaire surveys

As our study involved humans and medical studies, the research was performed in compliance with ethical principles of the Declaration of Helsinki and they were approved by the Research Ethics Committee of Kashan University of Medical Sciences, Iran (No IR.KAUMS.NUHEPM.REC.1401.004).

The cross-sectional studies were conducted in 2022 in the six finishing shops of carpet manufacturing industry in Kashan city, Iran. Participants included in the study were selected from 96 male workers exposed to VA in occupational working posts. Among the exposed group, 25 subjects were excluded due to diagnosed lung diseases including asthma, bronchitis, chronic obstructive pulmonary disease (COPD). Moreover, 17 workers who were physically or medically unable to conduct spirometry testing were also excluded from the investigations. In addition, 14 employees with a smoking history were excluded from the research as well. Ultimately, the VA exposed group consisted of 40 male employees with current respiratory exposure to VA with more than 2 years of work experience. As the control group, 40 males from the office personnel of the same manufacturing industry with approximately the same demographic characteristics and no history of inhalation exposure to VA or any other chemical compounds were selected.

The questionnaire surveys were performed based on the modified version of the American Thoracic Society (ATS) questionnaire (Ferris 1978). Face-to-face interviews were performed with the employees for collecting data on socio-demographics, past diseases, occupational history, tobacco smoking, family history, and occurrence of health symptoms like cough, phlegm, wheezing, breathlessness, chest colds and chest illness. All subjects involved in the study signed the informed consent before participating in the study.

### Sampling and determining occupational VA concentrations

Detremination of VA concentrations in the workers’ breathing zones of the finishing shops of carpet manufacturing industry were performed based on the Method 1453 of the National Institute of Occupational Safety and Health (NIOSH) (NIOSH 1998). Air samples from working posts were collected by adsorption method using solid sorbent tubes containing carbon molecular sieve (CMS) (160 mg/80 mg) made by Sigma-Aldrich, Germany. Altogether, 120 air samples were collected in the VA exposed group as for each employee 3 air samples were collected. In addition, 12 blank samples were colected. The sampling time was regulated from 70 to 120 min for each sample to control the breakthrough volume. At each sampling point, both sides of the sampling tube were broken and connected with the personal sampling pump of model AirChek TOUCH (5–5000 mL min^−1^, SKC Inc., Covington, NC, USA) with a flow rate of 200 ml min^−1^, and then connected to the collars of the participants vertically by a holder and a clamp. After sampling at each stage, the sorbent tubes were stored in boxes filled with dry ice to maintain the temperatures below − 4 °C and were transferred to the laboratory for analysis and quantification.

In the laboratory, the contents of the sorbent tubes in both the front (160 mg) and rear (80 mg) of the sample tube were transferred to 5 ml vials. Then, using the optimal NIOSH Method 1453 (NIOSH 1998), the samples were extracted by chemical desorption method and using 1 ml of methylene chloride (Sigma-Aldrich, Germany):methanol (Sigma-Aldrich, Germany) with ratio of 95:5 as a desorption solution. The samples of VA were exposed to ultrasonic waves for 30 min to complete extraction. In order to determine the amount of analyte, 1 µl of the prepared solution with split ratio of 1:5 was removed by a 10 µl syringe made by Hamilton (Hamilton Co., 4970 Energy Way, Reno, NV 89,502, USA) and at the injection port of a Gas Chromatography-Mass Spectrometry (GC–MS) (7890 gas chromatograph, and 5975 mass spectrometer, Agilent technologies, CA, USA) with a capillary column of the length of 30 m was injected. Helium gas was also used as a carrier gas with a flow rate of 2 ml min^−1^. The temperature of the detector was 260 °C, the temperature of the injection was 210 °C, the temperature of the column was 35 °C for 5 min, and after that, the temperature of the column reached 50 °C with a temperature change rate of 5 °C min^−1^ and hold for 1 min (NIOSH 1998). Finally, information about each chromatogram, including height and area below each peak, was extracted. In the present study, the Threshold Limit Value–Time Weighted Average (TLV-TWA) for VA vapors was 10 ppm (35.2 mg m^−3^). Given that the amount of TLV-TWA provided is assumed to be 8 h per day and 5 working days per week, in cases where the working hours per week were more than 40 h per week, the amount of TLV-TWA was corrected using the correction model of Brief & Scala (Zhang et al. 2018).

### Evaluation of spirometry indices and lung function capacities

Pulmonary capacity and volume comprising forced vital capacity (FVC), forced expiratory volume in the first second (FEV1), FEV1/FVC ratio and peak expiratory flow (PEF) were determined by a trained and skilled technician on site according to the standards of the American Thoracic/European Respiratory Societes using a portable calibrated spirometer (Spirobank II, Italy). The spirometer was calibrated twice daily with a standard 1 L syringe according to the relevant instructions. The mean of the predicted percentage of each of the lung function variables based on age, sex and race was calculated and estimated by a spirometer. Participants were asked to refrain from bathing for 2 h before the spirometry test. In addition, to make the participants more familiar with spirometry and related maneuvers, they were provided with the necessary training. Prior to the test, the subjects were sitting for 5 min, then were asked to stand in front of the spirometer in a normal, comfortable position and place a special clip on their nose. At least 3 acceptable tests were performed for each of the subjects and the best was selected from the 3 tests. Finally, the comparison of the results obtained was made between the occupational VA-exposure workers and the control group.

### Quality control and quality assurance (QC/QA)

Blank samples were taken both in field sampling and laboratory analysis to investigate the potential contamination and eventual errors in the process (i.e., sampling, transport, and analysis). The analyses revealed that the VA concentrations in each blank sample were lower than 1% of the values determined in the main samples. For the blank samples, the concentration values were deducted from the values of the target samples. Spike samples were applied to test the accuracy of the analyses and the recovery was equal to 91.16%. The calibration curve was performed by analyzing the standard solutions of VA at six different concentration levels, i.e., 0.5, 2, 10, 50, 100, and 200 μg ml^−1^ prior analysis of samples. Value of *R*^2^ for the calibration curve was 0.994. In addition, the limit of detection (LOD) and limit of quantitation (LOQ) for the compound in this study were 0.1 and 0.33 μg ml^−1^, respectively. Duplicate samples were measured and were determined within 5% of alteration.

### Non-carcinogenic risk assessment

After determining the exposure level of employees to the VA compound, the non-carcinogenic risk assessment was performed based on the USEPA methodology (USEPA 1989, 2009).

To assess the inhalational health risk first the amount of respiratory intake of the contaminant was calculated using the exposure concentration (EC) parameter values according to Eq. (1) (Delikhoon et al. 2018):

1 $$\mathrm{EC}=(\mathrm{C}\times \mathrm{ET}\times \mathrm{ED}\times \mathrm{EF})/\mathrm{AT}$$

where, EC is the exposure concentration (mg m^−3^); C, concentration of VA (mg m^−3^); EF, exposure frequency (days year^−1^); ED, exposure duration (years); ET, exposure time for receptor (h day^−1^); AT, averaging time (ED × 365 days).

**Fig. 2:**
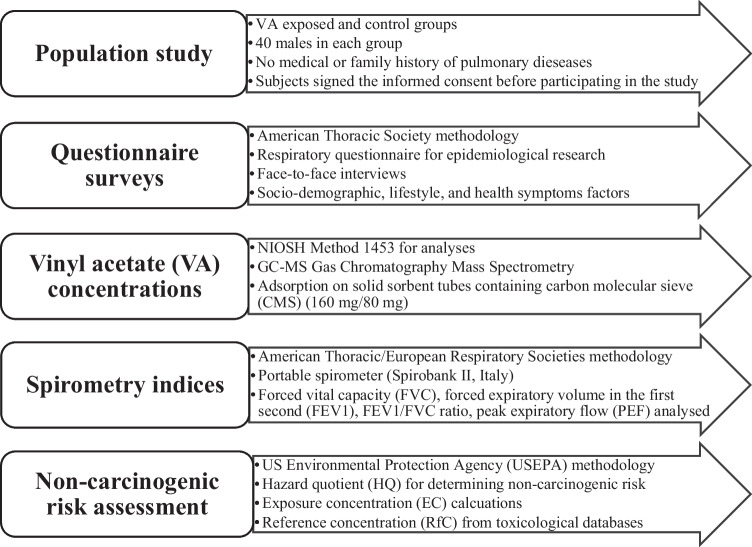
Conceptual diagram of the risk assessment analyses in this study

In the next step, to calculate the non-cancerogenic risk associated with inhalational exposure to VA, the hazard quotient (HQ) was used and calculations were performed according to Eq. (2) (Delikhoon et al. 2018):

2 $$\mathrm{HQ}=\mathrm{EC}/\mathrm{RfC}$$

where, HQ is hazard quotient (unitless); EC, exposure concentration (mg m^−3^); RfC, reference concentration (mg m^−3^). The exposure and toxicological parameters used for health risk calculations are presented in Table 1. The conceptual diagram of the risk assessment studies from occupational exposure performed in this research is given in Fig. 2.

**Table 1:** The exposure and toxicological parameters used in the health risk analysis

Parameter	Definition	Value	Reference
EF (days year^−1^)	Exposure frequency	300.00 ± 13.39	Questionnaire
ED (year)	Exposure duration	29.62 ± 1.53	Questionnaire
ET (hours day^−1^)	Exposure time for receptor	10.55 ± 1.82	Questionnaire
AT (days)	Averaging time	9000	USEPA 2009
RfC (mg m^−3^)	Reference concentration	0.2	USEPA 2022

### Monte Carlo simulation and sensitivity analysis

In conventional risk assessment methods, the risk is calculated and reported as a point estimate. Risk point estimation provides little information about the degree of uncertainty and variability around the estimated risk point (USEPA 1992).To address this issue, the USEPA recommends the simultaneous use of several descriptive variables in the technical guidance on risk assessment to obtain more accurate information on the value of risk or risk ratio. In this regard, the USEPA has introduced the Monte Carlo simulation method to risk assessment calculations (USEPA 1992). In the present study, the Crystal Ball software (version 11.1.2.4, Oracle Inc., USA) was used for the simulation and sensitivity analysis with 10,000 iterations.

### Statistics analysis

Data analysis was carried out by IBM SPSS Statistics Version 22 (Chicago, IL, USA). The data distribution was investigated by Kolmogorov–Smirnov test. Two-independent sample *t*-test and paired sample *t*-test were applied for comparing the mean of quantitative variables between the groups (case and control). Levene’s test was used for examining the equality of variances. Chi-square test was employed for evaluation of the frequency of respiratory symptoms prevalence between the case and control groups. In addition, for assessment of the relationship between inhalation exposure to VA and respiratory symptoms, logistic regression was used. The significant level in all tests was 0.05.

## Results

### Personal exposure at the workplaces

The results of the study population characteristics and respiratory exposure of investigated employees to VA in the carpet manufacturing industry are presented in Table 2 and 3, respectively. Between the values of demographic parameters in the exposed and control groups, no significant relationship was found (*P*-value > 0.05). The mean value of VA concentration in inhalation exposure in the exposed group was 12.73 ± 11.19 mg m^−3^ (3.61 ± 3.18 ppm) which was lower than the permissible exposure limit value (10 ppm) provided by the American Conference of Governmental Industrial Hygienists (ACGIH) but higher than the inhalation exposure limit (1 ppm) recommended by the National Toxicology Program (NTP) of the United States Department of Health and Human Services (US HHS) (Luttrell 2013). Furthermore, the VA concentrations in inhalational exposure were not detected in the control group.

**Table 2:** Demographic characteristics and respiratory exposure of employees to VA

Parameter	VA occupational exposed group (*n* = 40)	Control group (*n* = 40)	*P*-value
Age (year) ^a^	33.85 ± 9.81	34.37 ± 9.11	0.641^c^
Height (cm) ^a^	71.47 ± 13.64	70.75 ± 13.40	0.856 ^c^
Weight (kg) ^a^	174.40 ± 7.57	173.32 ± 7.32	0.764 ^c^
Body mass index (BMI) (kg m^−2^) ^a^	23.47 ± 4.21	23.54 ± 4.33	0.893 ^c^
Work experience (year) ^a^	9.40 ± 6.38	9.95 ± 5.81	0.610 ^c^
Working hours per day (hours) ^a^	9.25 ± 1.62	8.95 ± 1.35	0.374 ^c^
Educational level (%) ^b^			
Under Diploma Diploma Undergraduate Postgraduate	20 57.5 17.5 5	15 50 25 10	0.775 ^d^
Marital status (%) ^b^			
Single Married	17.5 82.5	20 80	0.639 ^d^
Vinyl acetate concentration			
mg m^−3 a^ ppm ^a^	12.73 ± 11.19 3.61 ± 3.18	ND ^e^	

^a^ Reported by Mean ± SD

^b^ Reported by percent (%)

^c^ Two-independent sample *t*-test

^d^ Chi-square test

^e^ Not detected

**Table 3:** VA concentrations (mg m^−3^) in the breathing zones of exposed group of employees

Working post	Range (mg m^−3^)	Mean (SD) (mg m^−3^)	N (%)
Brushing and beating (BB)	6.43–17.69	11.91 (4.06)	7 (17.5)
Sizing (S)	24.43–41.23	32.18 (5.60)	7 (17.5)
Shearing (SH)	7.41–24.83	15.92 (6.37)	8 (20)
Embroidery, zigzag, and rooting (EZR)	3.31–12.35	6.85 (3.63)	5 (12.5)
Quality control (QC)	0.07–0.25	0.16 (0.06)	5 (12.5)
Rolling and packaging (RP)	1.85–11.60	4.78 (3.00)	8 (20)
Total	0.07–41.23	12.73 (11.19)	40 (100)

### Respiratory symptoms and lung function parameters

The frequency of respiratory symptoms among case and control groups is presented in Table 4. The results revealed that employees with current inhalation exposure to VA had significantly higher prevalence rates of all investigated respiratory symptoms comparing to the non-exposed group. Also, the findings showed that occurrence chance for all symptoms were considerably higher in the case group in comparison with the control group. Moreover, a significant correlation was found between the prevalence of respiratory symptoms in exposed and reference groups (*P*-value < 0.05).

**Table 4:** Prevalence (%) of respiratory symptoms in exposed and control groups

Health symptoms	VA occupational exposed group (*n* = 40) N (%)	Control group (*n* = 40) N (%)	Odds Ratio	*P*-value ^a^
Cough	12 (30)	4 (10)	4.33	0.027
Phlegm	14 (35)	2 (5)	10.50	0.001
Wet cough	7 (17.5)	1 (2.5)	8.27	0.028
Wheezing	15 (37.5)	5 (12.5)	4.20	0.019
Dyspnea	14 (35)	1 (2.5)	21.00	0.0001
Chest tightness	7 (17.5)	1 (2.5)	8.27	0.028
Chest cold	16 (40)	1 (2.5)	26.00	0.0001

^a^ Chi-square test

Table 5 presents the findings of the evaluation of lung function variables. A significant difference was found between values of lung function test in case and reference groups (*P*-value < 0.05). Exposed employees have demonstrated lower levels of lung function parameters including FVC, FEV1, FEV1/FVC and PEF in comparison with the non-exposed group. As it is seen based on the values gathered in Table 5, all pulmonary function reference values decreased considerably in the exposed group.

**Table 5:** Pulmonary function measurements for exposed and control groups

Variable ^a^	VA occupational exposed group (*n* = 40)	Control group (*n* = 40)	*P*-value ^c^
Forced vital capacity (FVC) ^b^	80.37 ± 4.44	97.70 ± 9.97	0.001
Forced expiratory volume in 1 s (FEV1) ^b^	77.95 ± 4.22	100.82 ± 11.05	0.001
FEV1/ FVC ^b^	91.10 ± 5.15	103.72 ± 6.83	0.001
Peak expiratory flow (PEF) ^b^	79.97 ± 4.66	108.47 ± 16.29	0.001

^a^ % Predicted pulmonary function

^b^ Reported by Mean ± SD

^c^ Paired *t*-test

### Non-cancerogenic risk of occupational exposure to VA

The VA concentration values obtained in the inhalational exposure of the employees in the carpet finishing shops were applied to perform non-cancerogenic risk assessment. As shown in Table 6, the results revealed that the non-carcinogenic risk from VA inhalational exposure in all investigated posts of the process, except quality control (QC) unit, exceeded the acceptable level of one.

**Table 6:** Non-cancerogenic risk values (unitless) and probability distributions on different working posts

Working post	Range	Mean (SD)	Fractile				
			5%	25%	50%	75%	95%
Brushing and beating (BB)	5.31 × 10^0^–8.38 × 10^1^	2.74 × 10^1^ (1.14 × 10^0^)	1.29 × 10^1^	1.93 × 10^1^	2.57 × 10^1^	3.37 × 10^1^	4.90 × 10^1^
Sizing (S)	2.32 × 10^1^–1.50 × 10^2^	8.06 × 10^1^ (1.91 × 10^0^)	5.21 × 10^1^	6.66 × 10^1^	7.96 × 10^1^	9.26 × 10^1^	1.13 × 10^2^
Shearing (SH)	1.04 × 10^1^–7.99 × 10^1^	3.51 × 10^1^ (1.37 × 10^0^)	1.57 × 10^1^	2.39 × 10^1^	3.44 × 10^1^	4.44 × 10^1^	5.92 × 10^1^
Embroidery, zigzag, and rooting (EZR)	4.11 × 10^1^–2.99 × 10^1^	1.42 × 10^1^ (7.19 × 10^0^)	5.43 × 10^0^	7.94 × 10^0^	1.29 × 10^1^	1.94 × 10^1^	2.78 × 10^1^
Quality control (QC)	7.41 × 10^−2^–6.00 × 10^–1^	2.84 × 10^–1^ (1.10 × 10^–2^)	1.09 × 10^–1^	1.93 × 10^–1^	2.86 × 10^–1^	3.71 × 10^–1^	4.58 × 10^–1^
Rolling and packaging (RP)	1.32 × 10^0^–5.05 × 10^1^	8.83 × 10^0^ (5.51 × 10^–1^)	2.91 × 10^0^	5.13 × 10^0^	7.30 × 10^0^	1.10 × 10^1^	1.92 × 10^1^

The mean non-cancerogenic risk value in the whole carpet finishing process among investigated employees was equal to 2.93 × 10^1^ (SD = 3.46 × 10^0^). Analyzing the working posts separately, it was observed that in all exposure units, the average risk value exceeded the acceptable level (HQ = 1). The decreasing order of working posts based on the risk values (with the number of times the acceptable level given in brackets) is given as follow: S (80.6), SH (35.1), BB (27.4), EZR (14.2), and RP (8.83). Thus, it was demonstrated that relativley 95% of the risk results were above the acceptable value.

In RP post, the 5% fractile value of non-cancerogenic risk was 2.91 × 10^0^, which was above the acceptable health risk by 5%. In the QC post, the non-cancerogenic risk value in the 95% fractile was 4.58 × 10^−1^; thus, was below the acceptable health risk by 95%.

The probability and 5%, 50%, and 90% percentiles of non-cancerogenic risk values in all working posts were depicated separately in Table 7. In BB working post, non-cancerogenic risk values from VA exposure were 4.90 × 10^1^ and 2.74 × 10^1^ for 10% of the high-risk study population and for 50% of the study population, respectively. The non-cancerogenic risk even for 10% of the low-risk study population was 1.29 × 10^1^, which was 12-fold higher than the acceptable value (HQ = 1).

**Table 7:** The predicted probability of non-cancerogenic risk (HQ) of the respiratory exposure to VA for the employees of carpet manufacturing industry finishing shops in different working posts

Predicted probability of non-carcinogenic risk (HQ)	Carpet finishing process work posts					
	BB: brushing and beating	S: sizing	SH: shearing	EZR: embroidery, zigzag, and rooting	QC: quality control	RP: rolling and packaging
	Unitless					
P5 (5 percentile)	1.29 × 10^1^	5.21 × 10^1^	1.57 × 10^1^	5.43 × 10^0^	1.09 × 10^−1^	2.91 × 10^0^
Mean (50 percentile)	2.74 × 10^1^	8.06 × 10^1^	3.51 × 10^1^	1.42 × 10^1^	2.84 × 10^−1^	8.83 × 10^0^
P95 (95 percentile)	4.90 × 10^1^	1.13 × 10^2^	5.92 × 10^1^	2.78 × 10^1^	4.58 × 10^−1^	1.92 × 10^1^

### Sensitivity analysis

By applying Crystal Ball software, sensitivity analyses were performed to find the most crucial variables affecting the values of calculated risk. Table 8 presents the sensitivity analysis for the non-cancerogenic risks due to VA exposure. As it is presented in Table 8, the most crucial variable affecting the non-cancerogenic risks was the concentration of VA (C). The exposure time (ET) was placed second, followed by the exposure duration (ED).

**Table 8:** Non-carcinogenic risk (HQ) component share (%) in VA exposure in different work posts of the carpet finishing shops

Risk components	Carpet finishing process work posts					
	BB: brushing and beating	S: sizing	SH: shearing	EZR: embroidery, zigag, and rooting	QC: quality control	RP: rolling and packaging
	%					
VA concentration (C_VA_)	62.3	45.3	78.2	89.2	92.3	89.4
Exposure time (ET)	33.3	42.4	18.9	7.8	4.6	9.2
Exposure duration (ED)	3.0	7.6	1.5	1.9	2.3	0.8
Exposure frequency (EF)	1.4	4.7	1.4	1.1	0.8	0.6

## Discussion

This study aimed to determine the non-carconogenic risk from the inhalation exposure to VA among workers of the carpet manufacturing industry’s finishing shops. To the best of our knowledge, this was the first investigation assessing the human health risk of exposure to VA in any industry as well as evaluating the lung function and respiratory symptoms among employees exposed to VA.

Regarding the study population, the subjects in both groups: occupational VA-exposure and control, were characterized by the similar values of investigated socio-demographic parameters, namely age, weight, hight, educational level, and marital status. None of the employees chosen to the study population had the previous family or medical history related with pulmonary diseases. Moreover, the significant relationship between the values of analyzed demographic factors were not found both in the exposed and non-exposed groups (*P*-value > 0.05). The above indicates that the selected study population groups were relevant for further VA exposure investigations. Accordingly, the prevalence of respiratory problems and decreasing the reference values of lung function in this study as well as the risk values obtained in the risk assessment calculations can result from occupational exposure to VA.

Based on the GC–MS of occupational VA concentrations analysis, results obtained in our studies indicated that the mean inhalation exposure to VA among the exposed employees was 12.73 ± 11.19 mg m^−3^ (3.61 ± 3.18 ppm). Among all the investigated subjects, VA exposure was lower than the ACGIH’s permissible exposure limit (PEL = 10 ppm), but higher than the inhalation exposure limit (1 ppm) recommended by National Toxicology Program (NTP) (NIOSH 1998). Furthermore, inhalation exposure to VA was not detectable in the respiratory area of non-exposed employees.

The study of pulmonary symptoms in the current study showed that occupational VA-exposure employees had considerably more prevalence rate of all pulmonary symptoms including cough, dyspnea, wheezing, phlegm, shortness of breath, chest tightness, and chest cold in comparison with the subjects from the control group. Furthermore, the findings of logistic regression test revealed that after controlling the influence of confounding parameters, a statistically remarkable relationship between exposure to VA and the prevalence of all pulmonary symptoms (*P*-value < 0.05) was observed. Regression results demonstrated that inhalation exposure to VA incremented the risk of all pulmonary symptoms. The prevalence of pulmonary symptoms in occupational VA-exposure employees can increment the risk of pulmonary disease (Mirabelli et al. 2012).

The non-cancerogenic risk was estimated according to the reference concentration (RfC) value provided by the USEPA based on the toxicological studies. The mean non-cancerogenic risk (HQ) value in the whole production process among the investigated employees was equal to 2.93 × 10^1^ (SD 3.46 × 10^0^), which was 29 times higher than the acceptable risk level of 1. Based on the HQ values obtained for working posts separately, it was revealed that non-carcinogenic risk exceeded the acceptable value of 1 in the case of all posts, except quality control (QC) unit. The decreasing order of unacceptable risk on various working posts was the following: sizing > shearing > brushing and beating > embroidery, zigag, and rooting > rolling and packaging.

Sensitivity of the hazard quotient (HQ) distribution was determined against the uncertainty related to VA concentrations (C), exposure time (ET), exposure duration (ED), and exposure frequency (EF). The VA concentrations and exposure time appeared to have the most significant effect and contribution to whole uncertainty determined for finishing shops of carpet manufacturing industry. As it was revealed that the VA concentrations and the working time of the exposed employees had the highest impact on health risk values, thus it is recommended to reduce VA concentrations in occupational breathing zones and working time of exposed employees. Based on the values of the non-cancerogenic risk inhalational exposure to VA obtained, implementing engineering controls to decrease occupational exposure to VA is crucial.

Recent investigations have also shown that pulmonary symptoms related to VOC exposure can range from simple cough and irritation of the airway to lung cancer (Agarwal and Patil 2013; Goldizen et al. 2016). Exposure to VOCs such as VA can decrease pulmonary capacity. Evaluation of pulmonary function is more authentic than the questionnaire for the diagnosis of pulmonary diseases (Agarwal and Patil 2013). Previous investigations have shown that workers exposed to VOCs reduced their reference values of lung function (Wichmann et al. 2009; Kwon et al. 2018). The measurement of lung function parameters demonstrated a considerable difference between lung function parameters of occupational VA-exposure employees and the control group (*P*-value < 0.05). Occupational VA-exposure employees had lower levels of FVC, FEV1, FEV1-FVC, and PEF compared to the non-exposed group. The results of the present investigation indicated that inhalation exposure to VA causes long-term irreversible functional disorders in the respiratory system along with its acute and partially reversible effects.

Working in polluted workplaces such as the finishing shops of carpet manufacturing industry will increase the chronic respiratory symptoms along with gradual worsening of pulmonary function in occupational exposure employees to the pollutant (Hesam et al. 2020). These alterations are addressed in those employees exposed to pollutants such as volatile organic compounds (VOCs) in the breathing zone of the employees in different plants. Previous investigations (Wichmann et al. 2009; Hesam et al. 2020) have reported that inhalation exposure to VOCs causes stimulation in the respiratory tract, creates long-term pulmonary symptoms and decreases lung function variables.

Our study also has some limitations. As in the previous investigations (Wichmann et al. 2009; Scarselli et al. 2017) that have also reported that inhalation exposure to VOCs causes irritation in the airways and long-term pulmonary symptoms, the further investigations should focus on cumulative risk arising from cummulative adverse effects of various air pollutants (Pelletier et al. 2018).

Other limitation of our study was related with the investigated dispertion pathways of VA in the environment. Concerning distribution among various environmental compartments due to VA volatility, the most affected compartment is air (91.5%), and further aqueous environment (8.4%). VA may also enter the terrestrial compartment mainly by aerial deposition and in a small extent by spreading sewage sludge containing VA on soils. Photooxidation and wet deposition of VA and its transformation products are the main removal processes from the atmosphere. However, VA is not expected to bioconcentrate in terrestrial and aquatic organisms, to biomagnify in food chains, or to geoaccumulate (FIOSH 2008). Based on the above, as not all production and industrial application processes in the life cycle of VA were involved in the study, environmental risk related with air and water pollution is recommended to perform in the future.

Limitation in our study was also related with the investigated subpopulations. In our research on the VA exposure in the process of carpet finishing manufacturing industry, the study group involved only the male subjects. It might be the other limitation of our study as exposure among other subpopulations might vary. Especially, it would be recommended to extend the study on subsceptible subpopulations of children and pregnant women.

The attention towards child labor is still increasing. United Nations Organization through the International Labour Organization’s conventions (Minimum Age Convention No. 138 and the Worst Forms of Child Labour Convention No. 182) as well as the Sustainable Development Goals (SDGs) (i.e., Goal 8: Decent Work and Economic Growth) has provided the framework for national laws. Sadly, family poverty and lack of proper education are major drivers for children in low-income countries to be in the labor force (Khanam and Rahman 2008; Edmonds 2016; Sturrock and Hodes 2016; Keshavarz Haddad 2017).

In relation, as the birth rates are the highest in the low income countries (Pezzulo et al. 2021), there is a high probability that pregnant women are exposed to occupational hazardous factors with high frequency (Rahimi et al. 2020) that impacts female reproductive system, organ system, affecting hormonal impairments, molecular alterations, oxidative stress and DNA methylation, impairing fertility, and pregnancy (Kumar et al. 2019), as well as adversely affects the prenatal developmental of the fetus (Thulstrup and Bonde 2006). Moreover, as VA is a volatile compound, it might pose the risk for the customers who have bought these carpets. Therefore, inhalational indoor exposure in houses of the customers would be suggested in the future risk analysis.

Regarding exposure pathways only inhalation was investigated in our research. According to toxicological characteristic also dermal contact and adverse impact on eyes would be reasonable in future investigations. However, it would be also challenging as only a general human data on irritation caused by vinyl acetate are available (FIOSH 2008).

In accordance, in our research carcinogenic risk could not be calculated in accordance to the USEPA methodology. Due to the fact that at the moment VA is classified as possible carcinogen to human (Group 2B), no calculations could be performed due to the lack of the slope factor (SF) value in the toxicological databases. However, as VA is considered possible carcinogenic to humans, it can be concluded that in any case, the means of personal protection in working environments are highly recommended.

## Conclusions

The findings of the current study demonstrated that inhalation exposure to VA was of the high concern among male subjects investigated. The mean non-cancerogenic risk was equal to 2.93 × 10^1^ exceeding significantly the acceptable risk level of 1. The employees exposed to inhalational VA-exposure had considerably more prevalence rate of investigated pulmonary symptoms, including cough, dyspnea, wheezing, phlegm, breath shortness, chest tightness, and chest cold than subjects in the control group. Thus, engineering controls such as execution of proper local, dilution ventilation systems, and management controls such as decreasing the working time of employees and providing appropriate personal protective equipment are recommended to decrease the inhalation exposure risk of employees to VA. As these were the first studies on health risk and adverse affects of lung and pulmonary system due to the occupational exposure to VA, we also experienced limitations of the study, namely we investigated only inhalational exposure to VA solely in occupational exposure among male workers in the carpet manufacturing production process. Thus, based on the results of our preliminary studies, we would suggest including vulnerable subpopulations, especially children and pregnant women, as well as customers possessing carpets in their homes in the inhalational exposure pathway analyses, including dermal and eye exposure pathways in the risk analysis, and including other chemical compounds, like VOCs, for the cumulative risk assessment in further risk assessment research to provide more comprehensive risk characteristic related to VA exposure.

## Data Availability

The datasets generated during and/or analysed during the current study are available from the corresponding author on reasonable request.
